# The development of a machine learning algorithm for early detection of viral hepatitis B infection in Nigerian patients

**DOI:** 10.1038/s41598-023-30440-2

**Published:** 2023-02-24

**Authors:** Busayo I. Ajuwon, Alice Richardson, Katrina Roper, Meru Sheel, Rosemary Audu, Babatunde L. Salako, Matthew O. Bojuwoye, Ibraheem A. Katibi, Brett A. Lidbury

**Affiliations:** 1 National Centre for Epidemiology and Population Health, ANU College of Health and Medicine, The Australian National University, Acton, Australian Capital Territory, Australia; 2 Department of Microbiology, Faculty of Pure and Applied Sciences, Kwara State University, Malete, Nigeria; 3 Statistical Support Network, The Australian National University, Acton, Australian Capital Territory, Australia; 4 Sydney School of Public Health, Faculty of Medicine and Health, The University of Sydney, New South Wales, Australia; 5 Microbiology Department, Centre for Human Virology and Genomics, The Nigerian Institute of Medical Research, Yaba, Lagos State Nigeria; 6 Director-General’s Office, The Nigerian Institute of Medical Research, Yaba, Lagos State Nigeria; 7 Department of Medicine, University of Ilorin Teaching Hospital, Ilorin, Kwara State Nigeria

**Keywords:** Computational biology and bioinformatics, Microbiology, Medical research

## Abstract

Access to Hepatitis B Virus (HBV) testing for people in low-resource settings has long been challenging due to the gold standard, enzyme immunoassay, being prohibitively expensive, and requiring specialised skills and facilities that are not readily available, particularly in remote and isolated laboratories. Routine pathology data in tandem with cutting-edge machine learning shows promising diagnostic potential. In this study, recursive partitioning (“trees”) and Support Vector Machines (SVMs) were applied to interrogate patient dataset (n = 916) that comprised results for Hepatitis B Surface Antigen (HBsAg) and routine clinical chemistry and haematology blood tests. These algorithms were used to develop a predictive diagnostic model of HBV infection. Our SVM-based diagnostic model of infection (accuracy = 85.4%, sensitivity = 91%, specificity = 72.6%, precision = 88.2%, F1-score = 0.89, Area Under the Receiver Operating Curve, AUC = 0.90) proved to be highly accurate for discriminating HBsAg positive from negative patients, and thus rivals with immunoassay. Therefore, we propose a predictive model based on routine blood tests as a novel diagnostic for early detection of HBV infection. Early prediction of HBV infection via routine pathology markers and pattern recognition algorithms will offer decision-support to clinicians and enhance early diagnosis, which is critical for optimal clinical management and improved patient outcomes.

## Introduction

The need for improved access to early diagnosis and linkage to care has never been greater, with approximately 296 million people worldwide living with HBV, and approximately 820,000  people dying annually from HBV-related liver disease^1^. In a recent study we found the prevalence of HBV to be 9.5% (95% CI 8.1–11.0)^2^. As 90% of infected people are unaware of their infection status, and they are therefore at risk of infecting others^3,4^. The pathogenesis of HBV is characterised by different stages, each one with specific pathological characteristics and outcomes. Initial stages usually involve inflammation of the liver. Patients with persistent infection have an increased risk of progressive liver fibrosis, and life-threatening clinical complications of cirrhosis and liver cancer^1^. Therefore, early detection of HBV infection can assist clinicians in determining optimal timing for clinical management to prevent disease progression of infected patients.

Enzyme immunoassay is considered to be the gold standard for the diagnosis of HBV infection^5^. However, the widespread use of this specialised test in resource-constrained settings is limited, particularly for rural and remote laboratories, because it requires dedicated facilities, skilled lab technicians, and a continuous supply of electricity^6,7^. Nucleic acid test is also increasingly being valued for their diagnostic accuracy and clinical prospect in detecting the viremic stages of hepatitis B infection to guide treatment strategies, but the high-cost implication precludes its use for diagnosis in many clinical settings^5^. Consequently, there is a dire need for an accurate and reliable diagnostic technology to detect HBV infection earlier, without resorting to specialised immunoassay and prohibitively expensive nucleic acid tests.

Machine learning algorithms are adept at investigating medical phenomena by capturing complex and non-linear relationships in clinical data^8^. They form the basis of the digital healthcare revolution, the advent of which provides potentially important opportunities to advance innovation in medical research. A key feature that underpins the significance of machine learning in medical research is its potential to analyse large and complex data structures to create prediction models and design decision support systems. There is accumulating evidence that machine learning prediction models can assist clinicians to deliver personalised healthcare and drive a better future for patients^9,10^. For example, the first FDA-approved IDs-DR EyeArt^®^, an autonomous machine learning system that detects diabetic retinopathy in retinal fundus photographs, improved patient outcomes across multiple settings^11^.

Several other applications of machine learning to inform intelligent decision-making in healthcare have been cited in the literature. Breast tumours can be quantitatively diagnosed based on subtle morphological variations of myoepithelial cells with 90.9% accuracy by a machine learning algorithm^12^. Barakat and Bradley developed a predictive model to detect diabetes by a machine learning algorithm using features, such as sex, age and blood pressure^13^. Yip et al. developed a novel predictive model to detect non-alcoholic fatty liver disease in the general population by machine learning algorithms on the basis of 23 routine laboratory attributes^14^. Onu et al. developed a signal processing and machine learning enabled system to improve the diagnosis of birth asphyxia in low-resource settings^15^. Edeh et al. developed an ensemble learning model to predict viral hepatitis C^16^. Despite the proven usefulness of machine learning algorithms in these medical fields, the accuracy and reliability of algorithms in clinical practice continue to be debated. To increase the diagnostic efficiency and reliability, several studies in other populations have proposed the inclusion of more parameters from personal information to patient history and clinical examination^17^, and the use of feature selection to augment laboratory-based predictions^18^.

In this study, we investigate how machine learning algorithms can extract patterns in routine blood tests to detect viral hepatitis B, and we develop a diagnostic model of HBV infection for Nigerian patients. This model will enable early detection for those who live with HBV and will help provide greater access to care for vulnerable populations in resource–constrained settings, as well as support early intervention for rural and remote laboratories that do not have easy access to specialised immunoassays.

## Methods

### Ethics statement

All experimental protocols were approved by the Institutional Review Board of the Nigerian Institute of Medical Research (IRB/20/065) and the Human Research Ethics Committee of the Australian National University (2019/803), and conformed to the principles and guidelines outlined in the declaration of Helsinki. Patient data were anonymised. The Institutional Review Board of the Nigerian Institute of Medical Research and the Human Research Ethics Committee of the Australian National University approved the waiver of informed consent.

### Study setting

This study was conducted in Nigeria, a country with the largest population in Africa (estimated at 211.4 million in 2021)^19^, using patient data from the Centre for Human Virology and Genomics, Nigerian Institute of Medical Research (NIMR). NIMR is Nigeria’s foremost institute of medical research and hosts a dedicated HBV clinic.

### Study patients

De-identified data were extracted from patients who were suspected of HBV infection and subsequently underwent HBsAg immunoassay testing, between 2010 and 2020. A suspected HBV case is defined as a case that was compatible with standard clinical description, including elevated serum aminotransferase levels^20^. HBV patients co-infected with HIV or HCV and patients with any other infections were excluded. Immunoassay results were obtained from the GS HBsAg enzyme immunoassay platform (Bio-Rad, USA). The HBsAg response was classified as either "positive" or "negative" as dictated by specific NIMR reference intervals. All serum clinical chemistry analyses were performed using Cobas^®^ analyser, and haematological analyses were performed on the impedance colorimetric analyser (Bio-Rad, USA).

### Data pre-processing

Data pre-processing assigned each immunoassay case to HBsAg response category, with category 0 comprising HBsAg negative cohort, and category 1 comprising HBsAg positive cohort. Variables with greater than 65% missing data were excluded from further analysis, thus leading to the exclusion of the viral load attribute, as this was only available for a minority of the patients.

### Investigation of different subsampling settings

Subsampling was performed using up, down and random oversampling techniques to investigate whether imbalanced learning constitutes a significant problem^21^. These three methods were selected for their durability in medical literature to date, and their transparency for use in clinical data^22^.

### Development of a machine learning-based model

This study was conducted in accordance with the Transparent Reporting of a Multivariable Prediction Model for Individual Prognosis or Diagnosis (TRIPOD) reporting guideline statement checklist for prediction model development^23^. Prior to the machine learning analysis, continuous variables were summarised using mean and standard deviation, while categorical variables were summarised using percentage and numbers (proportions). The machine learning analysis was performed in R v3.5.1^24^, using the caret package^25^. The data set was divided into two parts, in a stratified train-test splits (70% training and 30% testing). Ten-fold cross validation was applied in the analysis to evaluate the performance of the predictive model. Two machine learning algorithms, namely recursive partitioning (“trees”)^26,27^ and SVM^28^, were used in tandem as classification algorithms and applied to patient data that comprised results for HBsAg (response variable) and routine clinical chemistry and haematology results (predictor variables). These supervised learning algorithms were used to ascertain predictor variable patterns and thresholds that differentiate HBsAg immunoassay positive from negative responses. Random forest algorithms, where the predictor variables (routine chemistry and haematology markers) were ranked in order of importance for classification as HBsAg positive or negative, were run on the patient data. The tree analyses, both forests and single decision trees provide an excellent precursor to SVM modelling, and were used to inform SVM modelling for the best predictors to include. The highest-ranked predictors from random forest modelling were applied to SVM for higher dimensional investigation (via kernel selection), to produce a final diagnostic predictive model for HBV infection. The C-classification method of SVM modelling was applied to the data set, and a radial kernel was used due to its applicability to data with complex features^28^. The machine learning interrogation included a tuning phase for each algorithm, to optimise the model hyper-parameters. Ten-fold cross-validation repeated ten times was performed with different hyper-parameter settings. We optimised the complexity parameter for decision trees, *mtry* parameter for random forest and sigma for SVM using tuneLength. R package caret was applied for the hyper-parameter optimisation. The optimal model was used to predict on the test data and predictions were compared to observed outcomes via a confusion matrix^25^. The predictive model was evaluated using the recommended performance measures for classification tasks^22,29^, including accuracy, sensitivity, specificity, precision, F1 and AUC. The R source code related to this study is available online at https://github.com/bia-ml/HepB-LiveTest.

### Web-tool development

To develop a tool that is amenable to use in clinical settings for prediction of HBV in real-time, a machine learning-enabled web-based app was designed, providing a Graphical User Interface (GUI) to access our final predictive model of HBV infection. This application is publicly accessible via https://www.hepblivetest.app/.

## Results

### Summary statistics for patient demographics

During the years 2010–2020, the final cohort for investigation comprised 916 individuals, with 59% male and 41% female. Age ranged from 10 to 89 years of age. The summary statistics for patient demographics are shown in Table 1. The reference interval and description of the 20 clinical attributes contained in the dataset are summarised in Supplementary Table S1. Sixty-nine percent of the patient cohort were HBsAg positive, thus producing a fairly imbalanced dataset. Age for HBsAg positive cohort generally incorporated a range of late-twenties to early-forties. Age was significantly different between HBsAg positive and negative cohorts (*t* = 13.54, df = 463, *p* < 0.0001) and a chi-squared test of independence showed a significant association between gender and HBsAg immunoassay response (χ^2^ = 20.51, df = 1, *p* < 0.0001).

**Table 1:** Summary statistics for patient demographics, Nigeria, 2010–2020.

Variable	HBsAg positive (n = 636)	HBsAg negative (n = 280)	*p* difference
Age	35.3 ± 10.7	47.0 ± 12.6	< 0.0001^a^
Sex	230 (36.2%) female	146 (52.1%) female	< 0.0001^b^
	406 (63.9) male	134 (47.9%) male	

*HBsAg* hepatitis B surface antigen.

^a^Two sample t-test, ^b^Chi-square test.

### Comparison between clinical attributes for patients testing positive or negative for HBsAg

**Figure 1:**
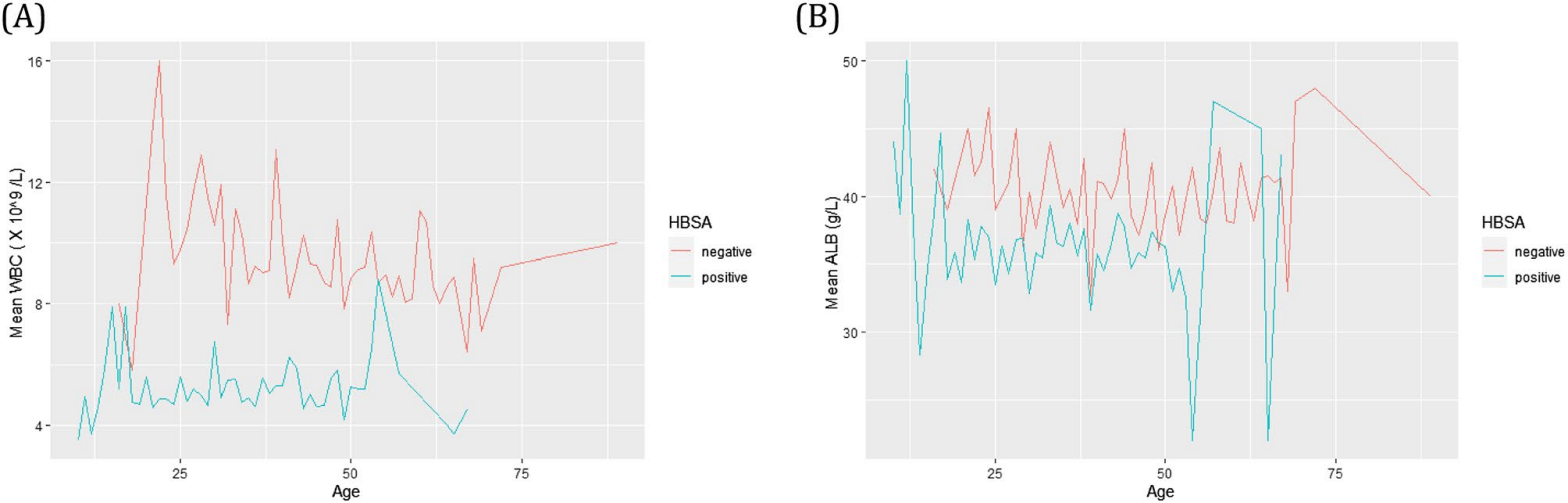
Kinetics for HBsAg positive (green) and HBsAg negative (red) cohorts across the age range investigated by SVM and tree-based machine learning algorithms. Comparison of mean WBC (**A**) and ALB (**B**) versus age at the time of testing.

Table 2 summarises the comparison between the clinical attributes of HBsAg positive cohort (n = 636) and HBsAg negative cohort (n = 280) and includes significance as estimated by unpaired *t* test. Routine markers that were significantly different were ALT, AST, GGT, ALB, and WBC. As expected, mean ALT and AST were higher for HBsAg positive cohort. But important to note was that WBC and ALB for HBsAg positive cohort had significantly reduced means compared to the HBsAg negative cohort for certain ages (Fig. 1).

**Table 2:** Mean serum and blood markers for patients testing positive or negative for hepatitis B surface antigen. Abbreviations for clinical attributes: ALT—alanine aminotransferase; AST—aspartate aminotransferase; ALKP—alkaline phosphate; Crea—creatinine; TBil—total bilirubin; GGT—gamma glutamyl transferase; ALB—albumin; Hb—haemoglobin; Hct—haematocrit; WBC—white blood count; PLT—platelet; MCHC—mean corpuscular haemoglobin concentration; MCH—mean corpuscular haemoglobin; MCV—mean corpuscular volume; RBC—red blood cell; RDW—red cell distribution width; Neut—neutrophils; Lymph—lymphocytes.

Clinical attributes	HBsAg positive (n = 636)	HBsAg negative (n = 280)	*p* value
ALT, U/L	111.9 ± 251.5	76.3 ± 146.8	0.0075
AST, U/L	87.9 ± 192.7	60.3 ± 117.8	0.0080
ALKP, U/L	84.1 ± 39.6	85.7 ± 41.4	0.5853
Crea, µmol/L	82.2 ± 39.6	89.1 ± 64.3	0.0973
TBil, µmol/L	16.8 ± 39.0	15.3 ± 24.5	0.4814
GGT, U/L	26.7 ± 17.3	30.2 ± 18.0	0.0063
ALB, g/L	35.9 ± 8.6	40.2 ± 6.0	< 0.0001
Hb, g/L	139.8 ± 19.0	138.8 ± 19.1	0.4650
Hct, L/L	0.4 ± 0.1	0.4 ± 0.1	1.0000
WBC, 10^9^/L	5.2 ± 2.0	9.3 ± 3.0	< 0.0001
PLT, 10^9^/L	252.5 ± 93.9	253.1 ± 89.3	0.9264
MCHC, g/L	340.5 ± 8.0	341.3 ± 8.4	0.1785
MCH, pg/RBC	30.3 ± 2.8	30.4 ± 2.5	0.5913
MCV, fL	89.0 ± 7.3	88.9 ± 6.4	0.8349
RBC, 10^12^/L	4.6 ± 0.6	4.6 ± 0.7	1.0000
RDW, %	14.1 ± 2.1	14.2 ± 2.0	0.4927
Neut, %	4.8 ± 4.4	5.2 ± 5.5	0.2830
Lymph, %	2.1 ± 1.0	2.2 ± 1.1	0.1933

### Tree analyses of HBsAg prediction patterns

**Figure 2:**
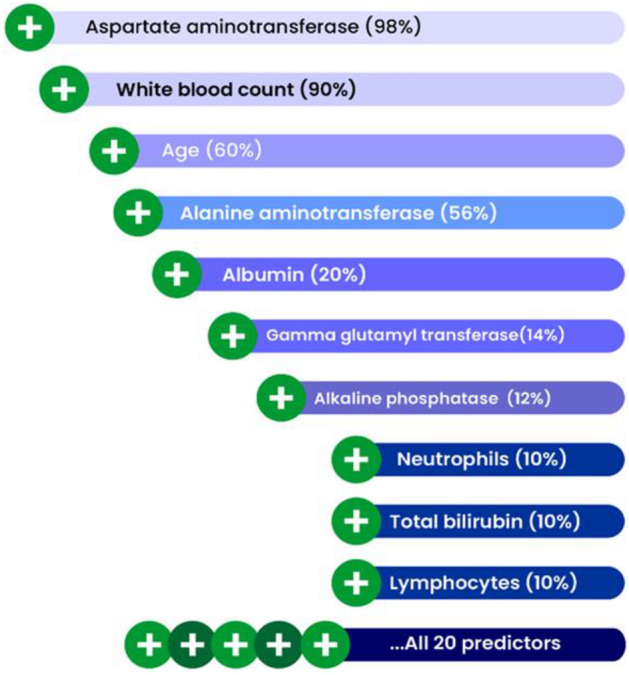
Variable importance from random forest analysis showing the leading predictors of HBsAg immunoassay results. The percentage in brackets represents the contribution of each predictor to the model performance.

Analysis by the tree-based machine learning algorithms provided a predictive model of HBsAg response. Random forest algorithms, where the predictor variables (routine chemistry and haematology markers) were ranked in order of importance for classification as HBsAg positive or negative, were run on the patient data. Overall, AST was the top-ranked predictive marker of hepatitis B infection in the Nigerian patient cohort, followed by WBC, patient age at the time of testing, and ALT. Albumin also appeared in the top five feature importance (Fig. 2).

**Figure 3:**
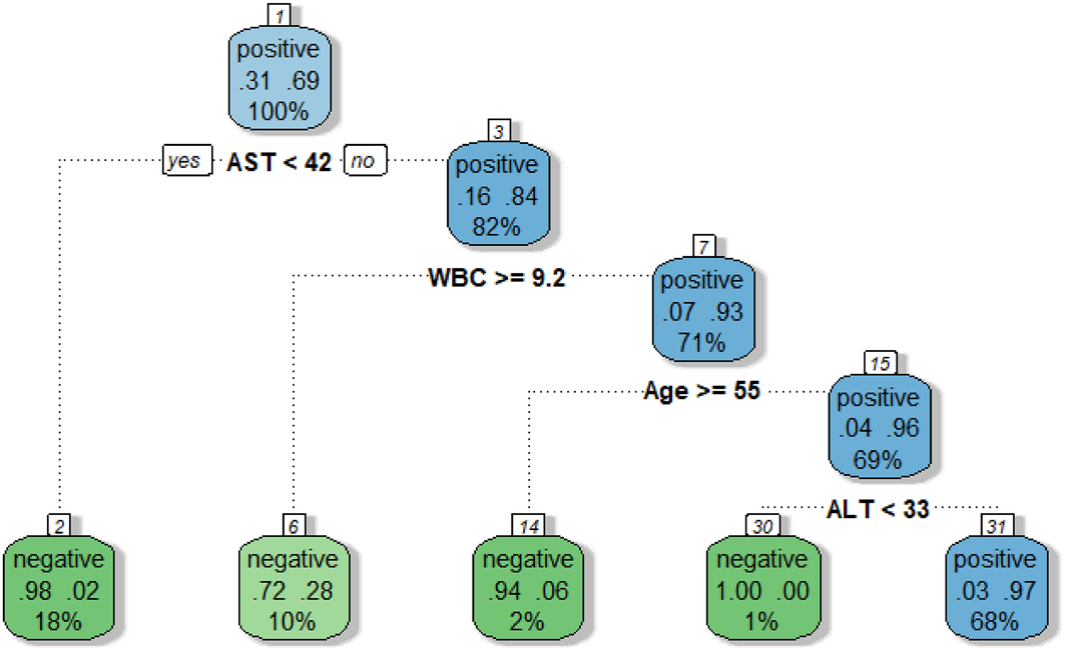
A decision tree exemplifying the analysis from random forest. Within the decision tree, predictor variable thresholds are calculated to formulate rules to guide HBsAg positive or negative prediction.

Figure 3 shows decision tree results for the same data used to produce the random forest summarised by Fig. 2. The advantage of single decision tree is the estimation of decision thresholds for each predictor used to understand a response, allowing the formulation of "rules" to define the classification accuracy of interest. Like random forests, classification accuracy is also calculated. Therefore, the following rule applies to the most accurate prediction of HBsAg immunoassay results;

$$\begin{gathered} AST < 42\;U/L + WBC > 9.2 \times 10^{9} /L + Age > 55\;years + ALT < 33U/L \hfill \\ \;\;\;\;\;\;\;\;\; = HBsAg\;Negative\left( {85.5\% \;accuracy} \right) \hfill \\ \end{gathered}$$

$$\begin{gathered} AST > 42\;U/L + WBC < 9.2 \times 10^{9} /L + Age < 55\;years + ALT > 33U/L \hfill \\ \;\;\;\;\;\;\;\;\;\; = HBsAg\;Positive \, \left( {96.2\% \, accuracy} \right) \hfill \\ \end{gathered}$$

Therefore, with four routine pathology markers, predictions of whether a patient has been infected with HBV can be made with an accuracy of 92.7%.

Results of the predictions were analysed via a confusion matrix (Table 3). Error rate was lower for the prediction of HBsAg positive classification at 3.8%, showing that the correct prediction of HBsAg positive immunoassay result was 96.2%. HBsAg negative classification prediction had higher error rates at 14.4% (suggesting correct HBsAg negative prediction at 85.6%). The metrics for measuring model performance are shown in Table 4.

**Table 3:** HBsAg immunoassay results classification error rate on test data for all patient cohort analysed by random forest (overall error rate = 7.30%). Table supports the results presented in Fig. 2. The top four predictor variables were used for the calculation of error rate.

HBsAg category	HBsAg negative	HBsAg positive	Error rate
Negative	77	13	0.144
Positive	7	177	0.038

**Table 4:** Performance metrics of random forest predictive model of HBsAg response.

Model performance	Prevalence (%)	PPV (%)	NPV (%)	Sensitivity (%)	Specificity (%)	Precision (%)	AUC	FI-score	ACC (Confidence interval)
	69.3	96.2	85.6	93.1	91.6	96.2	0.98	0.94	92.7 (95%CI: 88.9–95.4)

#### SVM analysis of HBsAg immunoassay results

**Figure 4:**
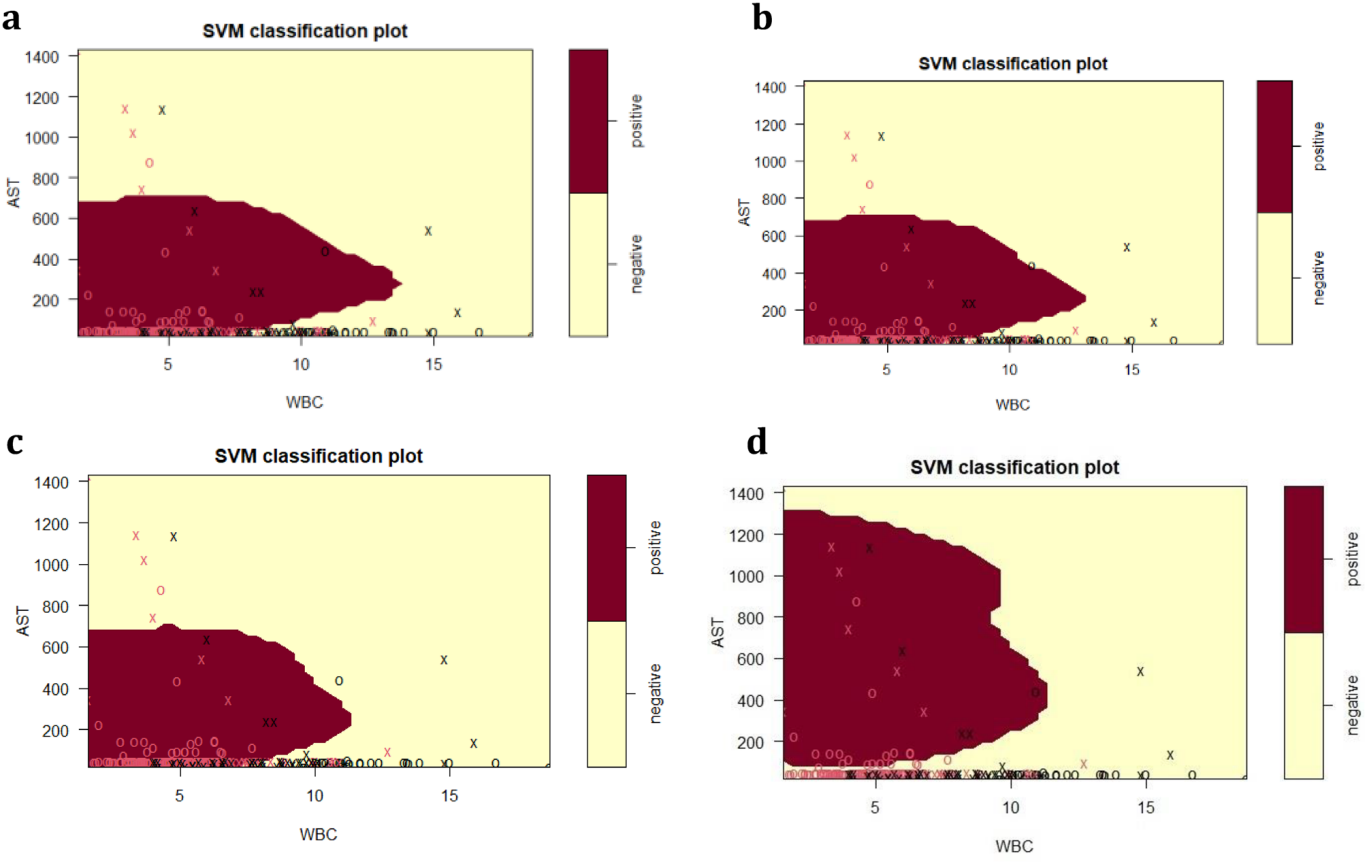
SVM plots describing the interaction of serum AST, WBC and serum ALT for the classification of HBsAg positive versus HBsAg negative results, as previously detected by specific HBsAg immunoassay. (**a**) ALT = 20 U/L; (**b**) ALT = 50 U/L; (**c**) ALT = 100 U/L; (**d**) ALT = 1000 U/L. The feature space assigned to HBsAg positive cases is shown in dark red, and the region assigned to negative cases is shown in light yellow. Crosses indicate the support vectors, and circles represent the remaining observations. The final SVM-based model of infection required to separate HBsAg positive from negative responses included the predictor (independent) variables AST (U/L), WBC (× 10^9^/L), age (years), ALT (U/L) and ALB (g/L): (cost = 4, gamma = 0.36, C-classification method and radial kernel). After tenfold training/testing of the data set, HBsAg positive immunoassay results were predicted at 88.2%, while negative results were predicted at 78.2%.

**Figure 5:**
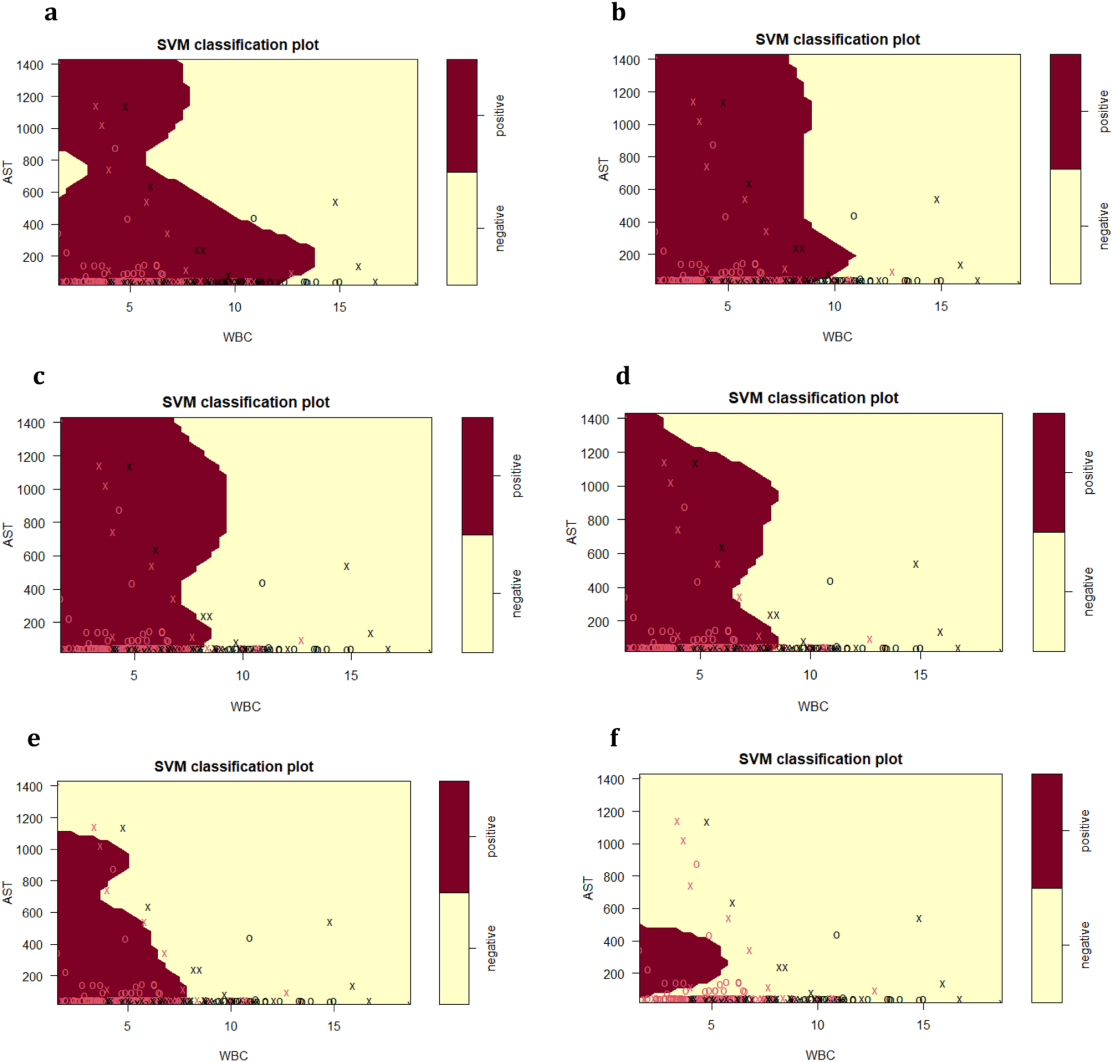
SVM plots describing the interaction of serum AST, WBC and patient age at the time of HBV testing for the classification of  HBsAg positive versus HBsAg negative results, as previously detected by specific HBsAg immunoassay. (**a**) Age = 15 years; (**b**) Age = 25 years; (**c**) Age = 35 years; (**d**) Age = 45 years (**e**) Age = 55 (**f**) Age = 65.

The final SVM-based diagnostic model of HBV infection represented by Fig. 4 and Fig. 5 predicted HBsAg positive results at 88.2%, while negative results were predicted at 78.2%, using the routine serum and blood markers AST, WBC, Age, ALT, as well as albumin. These top five predictor variables of HBsAg positive or negative immunoassay results from the tree analyses subsequently provided the model for calculations of prediction accuracy. The model was further assessed on accuracy (85.4%), sensitivity (91.0%), specificity (72.6%), precision (88.2%), F1-score (0.89), and AUC (0.90). The ROC curve showing the discrimination measure of the model is presented in Supplementary Figure S1. The relationship between AST and WBC were plotted for Figs. 4 and 5, with slices introduced into the models for ALT (20–1000 U/L, Fig. 4) and age at the time of HBsAg immunoassay testing (15–65 years, Fig. 5).

#### ALT kinetics associated with the primary predictor variables AST and WBC

For the SVM plots presented in Fig. 4, the dark red area represents the areas of HBsAg positive prediction, and the light yellow represents HBsAg negative prediction. A serum ALT of 20 U/L (Fig. 4a) is well below the upper limit of the NIMR reference interval, and therefore represents early phases of initial HBsAg positive infection, prior to liver damage. For Fig. 4a and Fig. 4b,c,d, a pronounced relationship between AST and WBC was detected, which interacted with ALT, as demonstrated by the alterations in AST–WBC relationship with increasing serum concentrations of this enzyme.

For Fig. 4 (a,b,c), while the upper range of AST stayed at approximately 700 U/L, two features of the HBsAg population (dark red) were pronounced with the increase of ALT from 50 to 1000 U/L. First was the decrease in WBC for the HBsAg positive category to almost 10 × 10^9^/L and the shrinking of the WBC concentration due to gradual increase in the upper limit associated with increasing ALT. At 1000 U/L ALT (Fig. 4d), the HBsAg positive category is defined by a higher serum AST range of 100–1300 U/L, and a further reduced WBC.

The SVM investigation summarised in Fig. 4 emphasise the interaction of ALT with WBC. An increase in ALT from 20–100 U/L resulted in a slight decrease in WBC for positive cases. With further ALT increases (1000 U/L), WBC decreases dramatically compared to 20–100 U/L. This may be an early warning of infection, particularly in individuals with suggestive histories. Within the ALT, AST, and WBC boundaries, additional decision support threshold can be estimated to allow the earliest possible detection of HBV infection, and this was achieved with only three routine markers.

#### Impact of age on the SVM prediction of HBsAg immunoassay result by AST and WBC

Figure 5 examines the impact of increasing age on the prediction of HBsAg immunoassay result by serum AST and WBC. The age range introduced into the SVM model was from 15–65 years at the time of HBsAg testing. As done for ALT (Fig. 5), the age factor was introduced into the SVM model as a static slice, hence providing a model of AST–WBC interaction at that specific age.

At 15 years (Fig. 5a), the diagonal pattern shape of the HBsAg positive class (dark red) dissects the class into two distinct sub-populations. The first sub-population is defined by higher WBC (0 to almost 15 × 10^9^/L), but AST concentration was less than 700 U/L, while the second sub-population is defined by a higher serum AST range of 700– 1400 U/L and a reduced WBC not exceeding 8 × 10^9^/L.

From 25 – 45 years (Fig. 5b,c,d), it was interesting to note that for the HBsAg positive class, the upper limit for serum AST remained consistent at around 1400 U/L, while the WBC reduced significantly from 11 × 10^9^/L to 8 × 10^9^/L, as the age increases.

Of particular interest for ages 55–65 (Fig. 5e,f) was the changing relationship between serum AST and WBC with increasing age and the associated shrinking of the HBsAg positive class. By 55 years of Age (Fig. 5e), serum AST level was reduced to 1100 U/L and a WBC of approximately 7 × 10^9^/L, while by age 65, HBsAg class is defined by lower serum AST range of approximately 50–500 U/L, with the WBC reducing drastically.

### Web-tool development

**Figure 6:**
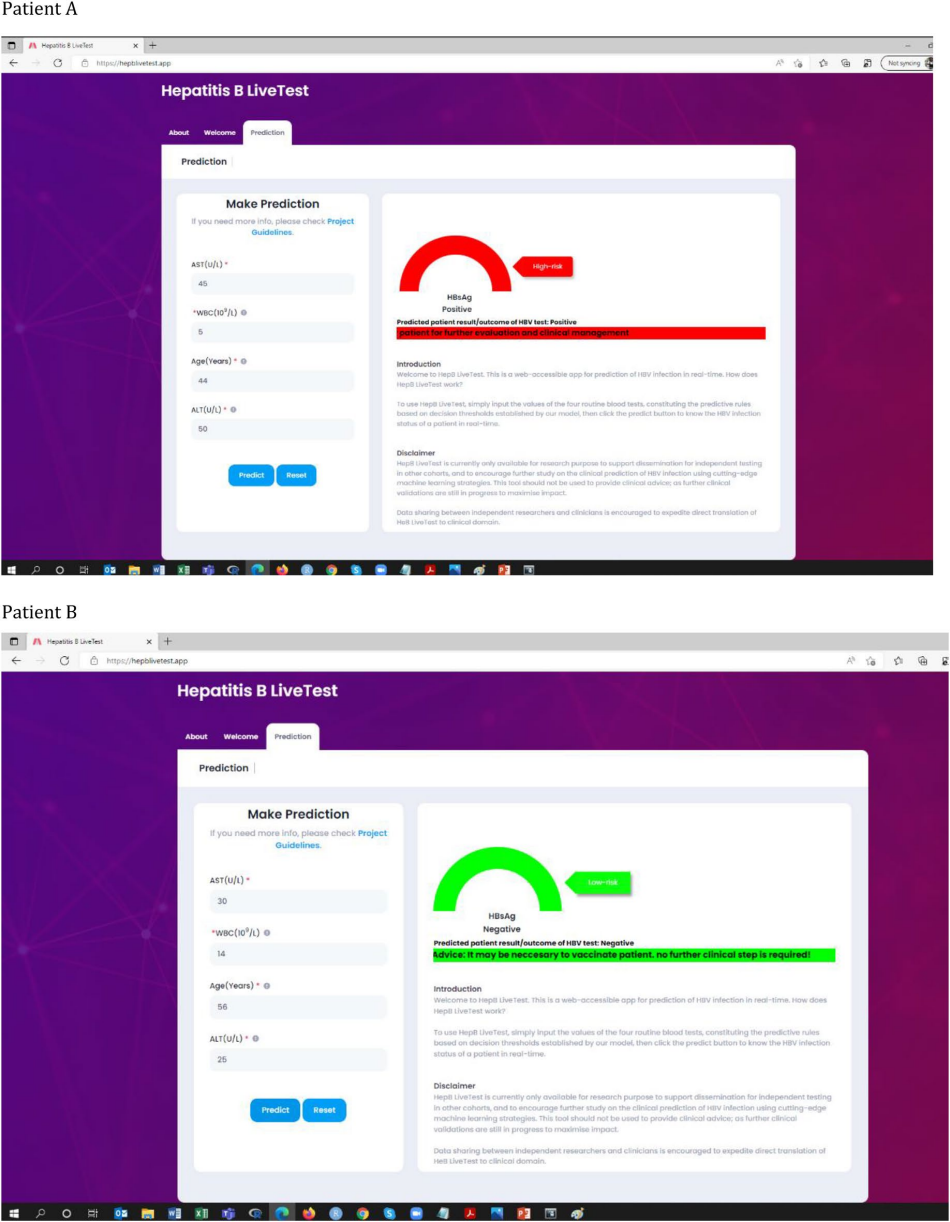
Web application of the model (https://www.hepblivetest.app/) and the result of two hypothetical patients (**A** and **B**).

To encourage further study on the clinical prediction of HBV infection status using cutting-edge machine learning strategies, our predictive model of HBV infection was translated into a free publicly accessible web-app as a decision support system (https://www.hepblivetest.app/). To use the machine learning-enabled web-app, referred to as HepB LiveTest, there is a need to input the values of the four routine pathology tests, constituting the predictive rules based on the established decision thresholds, followed by a click of the predict button to predict the HBV status of a patient in real-time. Figure 6 shows the web application of HepB LiveTest and the results of hypothetical patients.

## Discussion

Using tree-based machine learning algorithms (random forest and decision tree), and SVM, our study proposed a predictive model on the basis of 20 routine pathology and clinical attributes as a novel diagnostic model for early detection of HBV infection. The predictive model was assessed to have sensitivity 91%, specificity 72.6%, precision 88.2%, F1-score 0.89, and AUC 0.90, and thus rivals with immunoassay. This illustrates the potential of pattern recognition algorithms to enhance clinical decision support, facilitate diagnostic procedures, and improve patient outcomes—the enhancement pertains to early detection and savings of time, money and anxiety. All of these translate into significant cost savings for Nigeria’s health system and its citizens.

Machine learning interrogation of routine pathology data associated with HBV immunoassay results has previously been studied in other populations. Shang et al. investigated the interactions between HBsAg and other pathology markers in a Chinese patient cohort^30^. The study found that HBsAg immunoassay result can be predicted through combined Classification Decision Tree (CDT) and logistic regression modelling of associated predictor variables at 92.8% and 95% accuracy, with an overall CDT sensitivity and specificity of 94.7% and 89.5%, respectively. They found that the combination of ALT, ALB and ALP was the strongest predictors of HBV infection status in a Chinese patient cohort^30^. Whilst the high rates of prediction accuracy, sensitivity and specificity recorded in the Chinese patient cohort were matched in this study via random forest modelling, the predictive markers varied slightly in both populations. However, ALT and ALB were the most prominently featured predictive markers of HBV infection when comparing the profile of the Chinese patient cohort with the Nigerian patient cohort. Diagnostic markers and liver enzyme levels that are encountered in clinical settings may vary by geographical locations, populations and the ethnicity of the patients^31^.

The high prevalence settings of the Nigerian and Chinese study population may have contributed to the higher sensitivity and slightly reduced specificity values observed in these studies. This is consistent with available evidence in machine learning literature in healthcare domain^29^, where the objective is often to maximise the number of true positives and minimise the number of false negatives. This is particularly important to ensure that no positive case goes undetected— as the consequences of predicting positive patients as negatives (i.e. higher value of false negative) could have significant life-threatening clinical complications. Hence, models with higher sensitivity value is highly desirable, as it is not always possible to optimise sensitivity and specificity simultaneously. In a scenario where the objective was to maximise the number of true negatives and lower false positives, then the trade-off between sensitivity and specificity can be tuned by changing the threshold (cut-off point) to optimise specificity.

Serum ALT and AST are important enzymes for the clinical management of HBV infection, where severe elevations in content may be suggestive of a potential liver damage^32^. Of particular interest from our findings was that approximately 40% of HBsAg positive patients had an albumin level lower than the bottom threshold of the reference range, this suggests a sub-cohort of HBsAg positive patients with chronic liver damage. Serum albumin is produced specifically by the liver, and chronic hepatitis impairs the biosynthetic capacity for this liver function marker^33^. The value of WBC as one of the leading diagnostic predictors of HBV infection is consistent with a previous study^34^. In our study, the mean WBC was lower in HBsAg positive cohort, potentially suggesting that low WBC is an important indicator of infection, as patients with HBV infection may have low WBC^35^. Further, this study emphasised the importance of age as diagnostic predictor of HBV infection. The mean age of HBsAg positive cohorts was 35, this reflects the time of life when people are more likely to be involved in risky behaviours, such as having multiple sexual partners, and intravenous drug use, which could increase the risk of exposure to HBV, particularly in a hyper-endemic population.

Cross validation is a highly robust method for assessing model performance, and training the algorithm on 70% of the data, and testing on the remaining 30% is relatively robust to prevent overfitting^36^. Subsampling methods can improve model performance in the case of imbalanced datasets, but in our dataset, the application of subsampling methods had no impact on the model performance. Further, having scrutinised model metrics and appraised performance based on cross-validated prediction results, the risk of overfitting was found to be low. In addition, the minority class (HBsAg negative) count contains enough relevant dependencies to inform a classifier and accurately learn without significant disturbance from the imbalance. This usually takes ultimate precedence to the imbalanced proportions that may exist between minority and majority classes^37^. Further, it is important for models to be trained on a dataset whose distributions reflect the future real-world test cases for which they will ultimately be applied, particularly models with clinical applications. The classifier trained on balanced data may not be generalisable to real-world data that is naturally imbalanced, and would need to be re-trained on realistic data, for which predictions and patterns may change immensely^38,39^.

In our study, a tree-based algorithm, particularly random forest, enjoyed high classification accuracy and fast operation speed. Previous work has shown that random forest classifier outperforms hundreds of other classification algorithms^40^. Whilst a single decision tree is more interpretable than a random forest, the random forest algorithm has the ability to aggregate a large number of decision-trees using bootstrap resampling, and often yields lower variances and better model generalisation than single decision tree^41^. The SVM, on the other hand, does not discard cases like the tree-based algorithms that rely on node purity to predict a response classification^28^. Among the other advantages of SVMs, plotting the category patterns after applying the radial kernel produced visible evidential guidance on the nature of the classes being predicted. This was very useful when considering the AST–WBC interaction at different ages.

The results herein described were produced via blood test results only. Future investigations are necessary to combine routine pathology results with patient history and clinical notes to explore the prediction of HBV-associated disease outcomes. It will also be important to externally validate the model, particularly in settings with low prevalence of HBV infection, to inform evidence for generalisability and cross-site transportability. We have packaged our predictive model as a free, publicly available online tool to support its application for independent testing and validations in other cohorts.

In conclusion, the SVM model presented herein highlighted the utility of the serum AST concentration–WBC interactions to reveal predictive rules and patterns at varying serum ALT concentrations or age. With an eye to the future benefits to clinicians, hospitals and health systems, the predictive patterns described herein, once further validated in the field, could be integrated into existing computer systems in pathology department, to form intelligent systems in silico for the enhanced clinical management of HBV patients. Rules based on routine pathology data will power the intelligent system to identify patterns in patient clinical data, use the patterns to indicate early on whether the patient has been infected with HBV, and link them to care before their condition becomes worse, thus preventing the development of serious disease through timely interventions. This will significantly improve the current sub-optimal diagnostic and treatment rates for HBV infection in Nigerian population, and will enhance the world health organization’s targets of eliminating HBV as a public health threat by 2030.

## Supplementary Information

Supplementary Information.

## Data Availability

The authors declare that the data supporting the findings of this study are available within the paper and the supplementary information files. Raw data are available from the corresponding author in redacted form upon reasonable request. Correspondence and requests should be addressed to B.I.A.
