# Supplementary material for: The development of a machine learning algorithm for early detection of viral hepatitis B infection in Nigerian patients
Source: Sci Rep. 2023 Feb 24;13:3244. doi: 10.1038/s41598-023-30440-2 (PMC9958122; doi:10.1038/s41598-023-30440-2)
Supplement: Supplementary file 1 — Supplementary Information. [file 41598_2023_30440_MOESM1_ESM.doc]

**The development of a machine learning algorithm for early detection of viral hepatitis B infection in Nigerian patients**

Busayo I. Ajuwon^1,2*^, Alice Richardson^3^, Katrina Roper^1^, Meru Sheel^4^, Rosemary Audu^5^, Babatunde L. Salako^6^, Matthew O. Bojuwoye^7^, Ibraheem A. Katibi^7^, Brett A. Lidbury^1^

**Supplementary Table**

**Table S1.** Description of clinical attributes used in machine learning interrogation

| Clinical attribute Description and definition Reference range |
| --- |

| Response variables |  |  |
| --- | --- | --- |
| HBsAg | Hepatitis B Surface Antigen (marker of HBV infection)  Negative = 0, Positive = 1 |  |
| Explanatory variables |  |  |
| Age (years) | Patient (case) Age | Years |
| Sex (male or female) | Gender 0 = F, 1 = M | M or F |
| ALT, U/L | Alanine aminotransferase; an intracellular enzyme released after liver and other tissue cell damage | 10–50 U/L |
| AST, U/L | Haemoglobin | 10–50 U/L |
| ALKP, U/L | Alkaline Phosphate; found in liver, bone, intestine and liver | 21.3–106.3 U/L Adult |
|  |  | 21.3–213 U/L Children |
| Crea, µmol/L | Creatinine; excreted by filtration through glomerulus and tubular section | 44–115 µmol/L |
| TBil, µmol/L | Total Bilirubin levels are reflective of the rate that the body recycles the red cells in the blood; bilirubin is a breakdown product of old, spent red blood cells. | 3–25 µmol/L |
| GGT, U/L | Gamma-glutamyl transpeptidase; an intracellular enzyme also relevant to liver damage | 7–50 U/L |
| ALB, g/L | Albumin; major component of plasma proteins | 35–52 g/L |
| Hb, g/L | Haemoglobin | 100–175 g/L |
| Hct, L/L | Haematocrit; formerly known as “packed cell volume” | 0.41–0.50 L/L |
| WBC, 10^9^/L | White blood count | 4.5–11 x 10^9^ /L |
| PLT, 10^9^/L | Platelets; an agent in blood clotting | 150–350 x 10^9^ /L |
| MCHC, g/L | Mean corpuscular haemoglobin concentration | 330–370 g/L |
| MCH, pg/RBC | Mean corpuscular haemoglobin | 26–34 pg/RBC |
| MCV, fL | Mean corpuscular volume | 80–100 /fL |
| RBC, 10^12^/L | Red blood cell | 3.9–5.5 x 10^12^ /L |
| RDW, % | Red cell distribution width | 11.6–14.0% |
| Neut, % | Neutrophils; white blood cells, elevated by bacterial infection and early viral infection | 40–70% |
| Lymph, % | Lymphocytes; white blood cells, elevated by viral infection and some cancers | 20–50% |

| Abbreviations for clinical attributes: ALT—alanine aminotransferase; AST—aspartate aminotransferase; ALKP—alkaline phosphate; Crea—creatinine; TBil—total bilirubin; GGT—gamma glutamyl transferase; ALB—albumin; Hb—haemoglobin; Hct—haematocrit; WBC—white blood count; PLT—platelet; MCHC—mean corpuscular haemoglobin concentration; MCH—mean corpuscular haemoglobin; MCV—mean corpuscular volume; RBC—red blood cell; RDW—red cell distribution width; Neut—neutrophils; Lymph—lymphocytes. |
| --- |


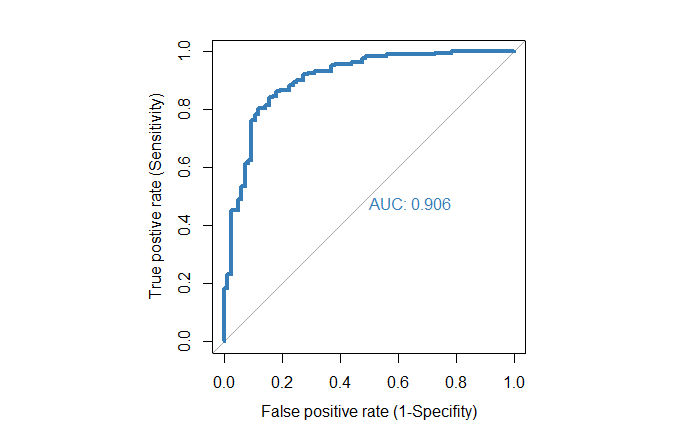


**Figure S1.** ROC curve showing the discrimination threshold of the machine learning predictive model
